# Meanings and practices of solidarity in global health: a qualitative investigation - study protocol

**DOI:** 10.1136/bmjopen-2024-095243

**Published:** 2026-01-07

**Authors:** Elysee Nouvet, Mary Ndu, Bridget Pratt, Gabriela Arguedas Ramirez, Barbara Prainsack, Unni Karunakara, Jantina DeVries, Samuel Asiedu Owusu, Caesar Atuire

**Affiliations:** 1 School of Health Studies, Western University, London, Ontario, Canada; 2 Health and Rehabilitation Science, Western University Faculty of Health Sciences, London, Ontario, Canada; 3 Australian Catholic University, Fitzroy, Victoria, Australia; 4 University of Costa Rica, San Jose, Costa Rica; 5 Department of Political Science, University of Vienna, Wien, Austria; 6 Global Health Justice Partnership, Yale University, New Haven, Connecticut, USA; 7 University of Cape Town, Rondebosch, South Africa; 8 University of Ghana, Legon, Ghana; 9 Oxford University, Oxford, UK

**Keywords:** Health Equity, Health policy, QUALITATIVE RESEARCH, ETHICS (see Medical Ethics)

## Abstract

**Abstract:**

**Introduction:**

Solidarity in global health is often invoked as an ethical imperative to guide responses to global health challenges. Its meanings and practices across diverse contexts, however, remain under-explored. Deepening an understanding of how solidarity is conceptualised, enacted and perceived by a diverse array of actors within the global health ecosystem is crucial to advancing meaningful and measurable application of this commitment in global health.

**Methods and analysis:**

This qualitative study uses interpretive research methodology to explore perspectives on solidarity among key global health stakeholders: community-level leaders in civil society organisations working on global health issues; research institute directors in the Global South; and individuals with experience of funding decision-making with major global health funding and agenda setting organisations (‘global health influencers’). Data will be gathered through semi-structured interviews and analysed using inductive and deductive reflexive thematic analysis, to identify patterns and differences in how these global health stakeholders recognise and define solidarity or its absence in their day-to-day work, while remaining attentive to conceptual tensions, participant interpretations of solidarity that may be unfamiliar to our team, and our role as researchers in shaping what we register and emphasise as significant in our reporting of findings.

**Ethics and dissemination:**

Ethics approval was obtained from the Western University Health Sciences Research Ethics Board (HSREB) in Ontario, Canada # 2024-123965-87873 and the Ethics Committee for the Humanities, University of Ghana # ECH 163/23–24 and University of Oxford, Oxford Tropical Research Ethics Committee (OxTREC) waiver dated 10 April 2024. Study results will be submitted for peer-reviewed publication. Results will also be summarised in an open access report and presented at various stakeholder meetings and in online webinars.

**Protocol registration:**

The final protocol was registered with Open Science Framework on 28 October 2023. View only link: https://osf.io/gryp5/?view_only=8baff435a35847f09a342408d38ee35b.

**STRENGTHS AND LIMITATIONS OF THIS STUDY**

- Qualitative methods are well-suited to generating detailed and nuanced description of how and why diverse global health stakeholders conceptualise and experience solidarity (or its absence) within their day-to-day work.
- The study’s interpretive design avoids treating solidarity as a concept with universal meaning, stressing the dynamic and situated nature of how this word and its significance for global health are defined.
- To the best of our knowledge, this is the first empirical global health study informed by elements of pluriversality, rendering available for discussion and debate what pluriversality-informed methods in global health research may involve.
- The study is not designed to and will not generate insight on regional or country-specific understandings of solidarity.

## Introduction

 Solidarity is a moral value and practice that expresses the idea of mutual support, cooperation and shared responsibility among people who face common challenges or risks.^1^,^3^ In the context of global health (GH), solidarity is, at times, seen as a guiding principle, goal and practice for addressing the health needs and inequalities of people around the world, especially those who are most vulnerable or marginalised.^4^,^6^

Solidarity in GH may take various forms, such as providing financial or technical assistance to low- and middle-income countries, based on an idea of fellowship among all countries, or all human beings; sharing knowledge and best practices; collaborating on research and innovation; respecting human rights and cultural diversity; and advocating for global justice and health for all.^7^ The spread of infectious diseases, antimicrobial resistance, climate change and the effects of globalisation are all threats that transcend national borders and have been identified in the literature as requiring solidarity and collective action to overcome.^8^ If we have learnt anything from the last pandemic, it is that no one country stands alone. The COVID-19 pandemic revealed the fractured nature of health systems worldwide, highlighting health discrepancies between the rich and poor, exacerbating GH disparities and emphasising the needs of the most vulnerable.^9^ It may be that solidarity represents not only a guiding principle but also a strategic necessity for ensuring the health and well-being of all. If this is the case, how can solidaristic action be promoted and measured across the GH ecosystem? And, in such a diverse ecosystem, how uniform or distinct are conceptualisations, experiences and practices of solidarity in and for improved collective health and well-being?

The idea of solidarity in GH, as we know it today, began gaining prominence in the late 20th and early 21st centuries.^10 11^ While calls for solidarity as ethical action have persisted within the GH ecosystem for decades, meanings of the word solidarity are unclear; the term is often inadequately or poorly defined. Moreover, many observe a failure globally to enact solidarity in the field of health. For instance, in the context of COVID-19, despite COVAX and the technology sharing platform C-TAP emerging with a promise of supporting equitable and timely access to COVID vaccines globally, it soon became evident that vaccine hoarding and nationalism superseded commitments to solidarity.^12^,^14^ Even though the governments of South Africa and India together led a global quest for a Trade-Related Aspects of Intellectual Property Rights (TRIPS) waiver for mRNA vaccine IP, governments in the global North actively blocked or refuted such proposals.^15^,^17^ Governments of countries such as South Africa were bullied into extractive contracts to purchase COVID-19 vaccines at higher cost than countries elsewhere. ^18^

Failures to enact solidarity globally are not accidental. Ours is an era of normalised tolerance towards structural injustices, linked to colonial and imperial legacies, and a dominant neoliberal capitalist world order.^19^ Globally, ethno-nationalism is on the rise, multi-lateralism in retreat, and foreign assistance programmes, most notably U.S. Agency for International Development (USAID) in 2025, are being cancelled. Political convictions rooted in a shared sense of humanity and common good conflict with the logic of neoliberal capitalism. The dominant ethos for political and economic engagements in this order is competition, not collaboration. Countries in the Global North and big corporations that profit from their dominance in this order do gain more, profit-wise and power-wise, by maintaining structural injustices and leveraging the business opportunities these afford, than working to transform them.^19 20^ It is not surprising against this backdrop that the rhetoric of GH solidarity often does not match the reality of GH governance and action. Despite frequent appeals to solidarity by world leaders and international organisations in the context of GH crises, such as the HIV/AIDS pandemic, the Ebola outbreak and the COVID-19 pandemic,^8 21^ no consistent mechanisms exist to ensure equity, justice and accountability in the production and distribution of health resources, such as vaccines, medicines, diagnostics and personal protective equipment.^1 8 9 13^ It is unclear to what extent those practices identified as key characteristics of GH solidarity in academic literature align with what others on the front lines of battles for justice, equity and accountability in GH regard as solidaristic, and regard as having realistic potential for uptake at the global level. If calls for solidarity or praise for solidaristic action are to have any meaning or ethically normative power in GH, what counts as a practice of solidarity, to whom, on what basis and with what necessary conditions for realisation must be clarified.

The study that we outline here has been designed in response to the tensions our team has observed between the rhetoric of solidarity by major players in GH, ongoing structures of injustice across the GH ecosystem and lack of evidence from those on the front lines of GH programmes and projects on what solidarity means and its potential for shifting injustices in that ecosystem. We use GH as a shorthand for efforts and initiatives aimed at improving health and well-being that are guided by justice principles (eg, equity, fairness), and informed by an understanding that addressing health challenges that impact multiple countries requires identification, understanding and action across borders.^22^,^25^ We will conduct and analyse ≅100 semi-structured interviews with three categories of GH actors: community-level leaders in civil society organisations (CSOs) working on GH issues; research institute directors in the Global South; and individuals with experience of funding decision-making with major GH funding and agenda setting organisations. This study is situated within a larger 5-year research project titled ‘Moving Beyond Solidarity Rhetoric in GH: Pluriversality and Actional Tools’. The broader study includes a series of scoping reviews and five international regional workshops aimed at surfacing under-heard perspectives on solidarity across the globe. Combining empirical research and critical interdisciplinary reflection on diverse engagements and conceptualisations of solidarity, a key output of this broader study will be a tool to guide GH funders aiming to translate their commitments to solidarity into action.

### Rationale

GH funders’ abilities to effectively reduce GH inequities are limited, when funding decisions are concentrated in the Global North, and based in understandings of how change happens that are not local to the worlds of applicants. It is widely accepted today that GH has its roots in colonial interests and paradigms.^26^ Allocation of resources and recognition within the sector continue to favour actors, institutions and knowledge from the Global North ^27^ . Decolonising or re-creating the sector requires acknowledging specific and interconnected forms of oppression, colonialism and domination that have excluded so many voices from debate and determination of GH agendas and approaches. 2628,30This exclusion has encompassed populations historically positioned on the peripheries of power and decision-making for GH policy and funding in general, and researchers in low- and middle-income countries and community-level actors in particular. Ensuring complex mechanisms of exclusion are acknowledged and actively interrupted constitutes key ethical commitments for GH action at this juncture. This requires new ways of working across diversely resourced regions, institutions and distinct social-cultural worlds and economically unequal contexts.

### Research question and objectives

Solidarity is currently one guiding principle that many have proposed can and should inform ethical GH practice.^2 6 31 32^ Organising ‘better’ or ‘good’” GH action around any principle requires clarifying what the value of that principle is in and for GH.

This original qualitative study aims to answer the research question ‘How do diverse GH actors understand, enact commitments to, and recognise solidarity or its absence in the GH ecosystem?’ To get to this question, we ask the following sub-questions:

- What meanings, associations and importance and effects, if any, does solidarity have for GH actors?
- What are conditions and indicators of solidaristic practices in GH, according to diverse GH actors?
- What factors account for any differences among GH stakeholders’ conceptualisations and commitments to solidarity?

In answering these questions, we aim to achieve the following specific objectives:

1. Enrich current conceptualisations of solidarity in and for GH. This will involve gathering and comparing various experiences, definitions, attributions of value to, factors associated with, and perceived threats or facilitators to solidarity in GH.
2. Generate a series of detailed examples of perceived solidarity in action and solidarity failures, observed by GH stakeholders within the context of their work to reduce global or local inequities. These examples will support achievement of objective 1, while enabling us to ensure conceptual additions remain grounded in localised examples.
3. Linked to the broader project on solidarity within which this qualitative study is embedded, identify considerations for the development of a tool to support solidaristic practice in and for GH.

## Methods and analysis

### Study design

This is a qualitative exploratory study that uses interpretive research methods,^33^ with implications for taking seriously and being transparent about the ways in which our own entanglements as researchers within the GH ecosystem at the core of this study may influence our analysis. Study design also reflects our interest in being attentive to historical inequities in GH knowledge production, decision-making and implementation that have left expertise from high-income countries over-represented, to the detriment of other perspectives. Fundamentally, we wish to augment acknowledgement and the potential for a plethora of perspectives on solidarity in and for GH. Our interest in foregrounding under-heard perspectives and in bringing diversely positioned actors’ views on solidarity into dialogue is informed by a conceptual framework, pluriversality,^34^ that is not at this point widely used in GH Research, to the best of our knowledge. We elaborate on the interpretive and pluriversal frameworks selected and their implications for study design below.

### Interpretive research design

We are adopting an interpretive research design,^33^ suitable to our intentions of exploring diverse participants’ accounts of what solidarity means and involves in their view, with attention to how these accounts may differ based on participants’ positions and experiences within a vast GH ecosystem. In accordance with the interpretivist paradigm at the core of interpretive research design,^35^ we are not approaching the main object of investigation in this study, solidarity, as something that can yield to fixed or universal representation. Instead, solidarity is regarded as a term whose meaning emerges in dynamic social processes within particular contexts and depends on interpretation by particular actors whose social identities, relationships to language, structures of power and biographically unique experience shape their understanding and application of this term.^33 35 36^

Flexibility sits at the heart of interpretive research design. Interpretive research design invites attention to emerging themes, unexpected interpretations and strives to expand understanding of social phenomena through a shuttling between existing theory and data.^35 37^ Reflexivity is also central to this design. Recognising that knowledge emerges within social processes and between distinctly situated human actors, this approach requires researchers to remain attentive to their role as co-producers of knowledge with study participants and critically consider the ways in which their own positionality shapes their interpretation and representation of participants’ narratives.^33 36^ More details on the research team’s positionality and implications for our study procedures are provided at the end of the Methods section.

### Pluriversality

The broader interdisciplinary study of solidarity in GH within which the present study sits is informed by a pluriversal approach. Calls for pluriversality in tackling GH challenges are relatively new.^1 38 39^ These calls align with and draw inspiration from calls to decolonise and indigenise GH, but also posit distinct requirements for escaping colonial ways of working that limit social transformation. One of these is that in bringing diverse perspectives into dialogue to tackle GH challenges, we actively resist modernist, colonialist norms of asserting universals and binaries that oversimplify or eclipse worlds and ways of being and acting that do not fit into such models. This will be the first qualitative GH study to the best of our knowledge that attempts to translate this element of pluriversality into research practice.

The implications of a pluriversal approach for qualitative research, in our understanding, include (but are not limited to) the following key components: (1) surfacing perspectives that have been historically silenced; (2) sampling for optimal diversity, rather than saturation; (3) creating opportunities for, and being attentive to, expressions of desired worlds, rather than limiting exploration and analysis to needs and problems in existing social order; (4) avoiding forms of representing findings that erase diverse ways of being, doing and knowing through construction of consensus; (5) practising critical reflexivity to avoid imposing assumptions about what descriptions or experiences mean, based on one’s own positionality. A pluriversal framework aligns well, in our view, with tenets of interpretive research design, specifically its openness to unexpected themes, and attentiveness to the ways in which meaning-making is contingent on the situated lens of those engaging in acts of representation, whether that be responses in interviews or the write-up of research findings.

Pluriversality aligns with our team’s convictions that the transition to a better, more sustainable and inclusive world cannot be accomplished by relying on a single worldview or way of thinking. The concept of the pluriverse was powerfully described by Latin American social (re)design and post-colonialist theorists.^34 40 41^ At its core is the assertion that while we may all as humans occupy one universe, we inhabit multiple worlds. Denial of that multiplicity is rooted in colonial and modernist paradigms that have served to delegitimise and violently erase Indigenous and non-Western ways of being and knowing. This denial of multiple worlds has also limited social change, in its contingency on pushing out of visibility and consideration not only intertwined mechanisms of colonialism, oppression and domination, but also vast and potentially important alternative ways of being, understanding, collaborating and (re)imagining what is possible.

A pluriversal-informed qualitative research design does more than recognise the variety in ways in which exploitation, oppression, exclusion and colonialism have taken grip in different places, or insist on the importance of respect for, and inclusion of, diverse and historically subaltern perspectives to effectively tackle challenges. In Leitão’s interpretation, research geared to supporting a Pluriverse—a world where many worlds co-exist and flourish—also requires shifting research geared to social transformation from a traditional focus on what needs to change in the current system, to a focus on desires. As Leitão notes, we need to foreground desires for possibles beyond what exists, if we are ‘to create and nurture alternative ways of world-making and contribute to the construction of other worlds’.^42^ Conducting research premised on and supporting a Pluriverse means being attentive to visions of the possible beyond what is, and ideally avoids romanticising certain groups or perspectives as inherently more legitimate for informing social transformation.

Pluriversality asserts the possibility of collaborating across differences, without erasing the complexity or significance of those differences. So it is that, for example, in indigenous enactments of pluriversal social (re)design described by Escobar, social groups with diverse interests and values manage to come together respectfully and achieve social projects for living better, in ways that balance competing priorities and avoid creating new hierarchies.^34^ The goal in analysis using this theory is not a consensus. It is, in our understanding, about bringing a broad range of perspectives into dialogue and debate, in order to identify where the points of resonance and opportunities for effective collaboration may lie. If goals to a problem are to be proposed in some common language, the limitations of this common language must be explicitly acknowledged. Moreover, and echoing core ideas of deliberative democracy,^43 44^ space must be left for the ethical validity, perceived necessity and pragmatic strategies of those bringing these goals back to their unique context, adapting these in form and meaning to the social, cultural, environmental or other relevant particularities of that context.^45^,^47^

We are aware that centring under-recognised and in particular community-level experiences, meanings and ways of working is insufficient in and of itself to render solidarity more actionable at a global level. As noted in the introduction, GH work exists within a dominant world order premised on competition and hierarchies of power, entrenched through colonialist and imperialist legacies and organised to reproduce itself, inequities and its ways of working. If insights from this study are to impact on practice at the global level, our team’s view is this requires buy-in from big players in the ecosystem. This underlies our decision as a team to make a key output of the broader research programme within this study a tool to guide GH funders aiming to translate their commitments to solidarity into funding policies and practices.

### Data collection

Data collection for this study will involve one-on-one semi-structured interviews with diverse actors across the GH eco-systems. Interviews are well suited to gaining detailed and nuanced insight into ways of thinking, being and acting that are central to the surfacing of diverse worlds we are seeking. We are recruiting approximately 100 participants, falling into three main categories: (1) community-level leaders in CSOs working on GH issues, (2) research institute directors in the Global South and (3) individuals with experience of funding decision-making with major GH funding and agenda setting organisations (‘GH influencers’).

Deciding on who to seek out for interviews, and whether or not to actively recruit different types of actors in the GH ecosystem, was not without debate. Some on our team felt strongly that our pluriversal framework should translate into centring insights and experiences of historically and conventionally excluded or lesser heard GH actors. Disrupting norms of GH discourse that reproduce the authority of certain worlds and knowledges while excluding others requires intentionally seeking out and asserting the validity of alternative perspectives. Based on this rationale, all participants might be recruited from among a category of actors that conventionally holds limited power over GH fundings and other discourses: actors working on GH issues at the community level. Ultimately, a decision was made to expand recruitment beyond this basis for inclusion, based on two logics. First, hearing from both community-level actors and actors who do not work primarily at the community level might render more apparent important particularities to ways of thinking about and enacting solidarity, depending on whether participants worked primarily at the community or non-community level. The insights from community-level participants might thus become more apparent to us as a research team and easier to describe in our analysis, through comparison to empirical data from interviews with otherwise positioned GH actors. Second, it was agreed that our team could not and should not ignore that GH research institute directors and major funding agencies hold central (though certainly distinct) roles in enacting or interrupting norms of practice in the sector. Gathering, analysing and rendering available for broader consideration reflections on solidarity from these actors might illuminate important considerations for rendering community-level actors’ insights on solidarity more actionable at non-community levels.

Another key sampling decision centred on participant inclusion/exclusion based on language fluency. Given our pluriversal framework, and desire to centre under-heard perspectives, we understood the importance of offering to participants the opportunity to participate in interviews in the language of their choice. At the same time, the PI (EN) was concerned about her ability to interpret participant meanings accurately for interviews conducted in languages in which she is not fluent (Spanish, English, French), as well as concerns about our team’s capacity to verify the accuracy of translations beyond the eight languages we master as a team. In the end, a decision was made that we would offer to conduct interviews in the language of participants’ choosing, with plans to appropriately resource high-quality professional interpretation and transcription/translation. We also will acknowledge in publications the limitation of our analysis where/if we are relying on translations of interviews from languages in which the team is not fluent. Sampling and recruitment strategies for each of our three participant categories are described below.

#### Community-level leaders

A majority (≅60) of those interviewed will be community-level leaders CSOs working on GH issues. Ensuring the voices of community-level participants are integral to our goals of being particularly attentive in our exploration to the perspectives of actors in the GH eco-system who have had limited power over GH funding and agenda-setting. For this first category of participants, we are prioritising recruitment from organisations, associations and movements whose work is guided by social justice principles, focused on improving health and well-being, and concerned with challenges to health and well-being that they identify as linked to transnational factors, situations and/or impacts. To be eligible for inclusion in this group, participant organisations must identify as either CSOs, community-based organisations or locally rooted non-governmental organisation. They also must be explicitly guided by justice principles (as shown through their public facing communications), must work with other groups on a regular basis to achieve their goals and must have emerged and continue to operate in the context where they were formed (even if they also work beyond that context).

The research team will compile an initial list of eligible organisations through searches of regional and international CSO directories and health advocacy networks. Sampling within this category of participants will be purposive^48^ and aim for optimal variation, rather than saturation. Reaching saturation in qualitative research implies an assumption or prediction that further data collection would reinforce what has already been established in a sample or sub-section of a sample.^49^ We make no such assumptions. Our sample size is pragmatic based on feasibility and aligned with objectives of expanding understanding of what solidarity is, means and could do for GH by multiplying insights on this topic, with an intentional recruitment and consideration of perspectives beyond the usual contributors to dominant GH discourse. All participants in this category will be in a policy, implementation or evaluation leadership role within their organisation and may be serving in either a paid or unpaid capacity (volunteer). We will aim to recruit participants across all WHO regions, and from diverse countries within those regions, but we will not be interpreting or comparing participant responses based on their location at birth or at time of interview. Indeed, such analysis would almost certainly involve making very problematic assumptions, given our sample size, about how participant’s perspectives on solidarity connect to regional or national influences. We are recruiting across WHO regions and diverse countries towards maximising potential for variation in participant perspectives. Again, towards achieving optimal variation, recruitment will be attentive to achieving diversity in terms of participants’ area of focus, with target recruitment of an equal number of participants working in each of the following seven areas: environmental justice, migrant and refugee issues, women and gender-based violence, access to medicine and treatment, indigenous rights, environmental justice and anti-poverty movements.

Initial contact will be made through email, in English by the interview team lead (MN), or by another member of the interview team fluent in French or Spanish for potential participants in countries where those languages are the main language of business. Where direct potential participant email contact is not available, we will use general organisation emails and listed phone numbers, or messaging through the organisation’s LinkedIn page to seek out contact information for leaders in the organisation. In a method used for all participant categories, initial contact via email will involve briefly describing the study, sending a one-page description of the project and offering to send the letter of information and consent with more detail should participation in an interview be of potential interest. If no response is received, 14 days after this initial contact, we will send a follow-up email. If a potential participant does not respond through these efforts, we will move on to another potential participant.

#### GH research institute directors

Partnership and collaboration between high and low- and middle-income country (LMIC) researchers is integral to developing best practice in GH policy and practice. The limits to equity in partnerships and collaborations within GH research that bridge Global North and South are well documented.^50^,^52^ Given this, we will recruit 15 participants (n=15) with leadership roles in GH research institutes located in and dedicated to work in LMICs. These participants will be recruited from university-funded, government-funded and privately funded research institutes as well as collaborative research networks. We will identify participants through a combination of web searches of research institute directories, review of collaborative networks (eg, Alliance for Health, Policy and Systems Research and regional research consortia). Research institutes will be purposively selected to ensure diversity in geographic region, institutional funding model and area of health focus. Eligible participants must hold managerial, senior research positions or an equivalent leadership position in their institutes. Inclusion of these participants will be purposive but also opportunistic, drawing on our team’s awareness of the GH research institute landscape for contacts, while prioritising potential participants working in diverse settings across the Global South.

Where direct email contact is unavailable for a research institute director and potential participant, we will contact the research institute directly to seek the email address needed for contact. As per our methods for community-level participants, potential interviewees will be contacted by the lead researcher (MN) unless contact is to be made in French or English, in which case another member of the interview team with that language fluency will assume responsibility for contact.

#### Influencers

We are not limiting our data collection to historically underheard and under-solicited perspectives. We are open to identifying frictions as well as points of alignment in understanding solidarity from across the GH ecosystem. We also recognise that for any of our research to impact policy and practice, its objectives, content and outputs must be legible and deemed valuable across the GH ecosystem, including by those who control funding and decision-making power in the sector. Our recruitment of ≅25 GH ‘influencers’ reflects this thinking. These actors are being recruited from major GH funding and agenda-setting organisations (‘GH influencers’). Inclusion criteria for these organisations are determined by systematically scoping top public and private funders and implementers in GH using the OECD report and other GH sites, that is, Donor tracker. In order to be eligible for participation as part of this group, an actor must have experience as staff in that organisation in the past year. As a team, we will discuss the list to ensure diversity of representation across donor types, regions and institutional mandates.

We will exclude from this ‘influencer’ category any participant from organisations that do not meet our inclusion criteria. However, we will also exclude participants from organisations that seem duplicative of those we already selected to participate—multiple organisations may have similar functions and profiles, and should this case arise, we will choose one in the interests of diversity. We will also exclude organisations that have historically been involved in unresolved disputes over conduct on funding, implementation, interaction with indigenous communities, as well as organisations whose main objectives are profit or political gain. Excluding them will ensure the integrity of the research. As per the approaches used for the two other participant categories, initial contact will be made via email by the lead interviewer (MN) or another member of the team should contact in a language other than English be deemed more appropriate. Follow-up emails will be sent 2 weeks after initial contact as needed. Where participants do not respond in the stated period, the PI and MN will identify alternative participants from the approved list of organisations.

### One-on-one semi-structured interviews

Interviews will be led by a team of interviewers, located in several countries and supervised by the principal investigator, an experienced anthropologist and qualitative researcher. All interviewers will complete ethics training and interview training before commencing and receive feedback and guidance on initial interviews from the PI. Interviews will be completed primarily over Zoom, though in rare instances, where the opportunity is present due to the presence of a researcher in the same location as a participant, a face-to-face interview will be offered as an alternative.

After signing the informed consent form, participants who agree to an interview will be asked to meet with an interviewer at a time convenient to them. Participants will also be made aware that should they require specific accommodation (eg, due to religious observance or disability), we will consider the best way to support their participation, in consultation with them. Interviews will be conducted in the language of choice of the participant and digitally recorded with the participant’s permission. If an interpreter for the interviewer is required due to a participant’s language of choice being other than one in which our team holds fluency, consent for this approach will be sought from the participant ahead of time and confirmed at time of interview. If a person wants to participate, but does not want to be recorded, notes will be taken on the interviewer’s laptop or tablet during the interview.

Interviews are anticipated to last between 60 and 120 min. All recorded audio from interviews will be saved on password-protected tools and conserved on Western University’s One Drive. Only study investigators and interviewers will have access to these. Interviews will be transcribed verbatim in the original interview language of the interview, with transcripts checked for accuracy and completeness by a member of the team. A third-party certified translator service is completing translation into English of non-English transcripts. The quality of translations is being verified by a member of the team with the appropriate language fluency.

### Data collection tools

The semi-structured interview guides, distinct for each category of participant (see onlinesupplemental files AC), consist of 10 open-ended questions with probes and are designed to surface definitions, practices and perceived barriers, facilitators and impacts (power, importance, relevance) of solidarity in and for advancing equity and social justice in GH. Participants are invited to share their understandings of solidarity and any aspirations they have for ways of working better across differences within or across organisations. A significant portion of the interview is dedicated to hearing participants’ examples of solidarity in practice and solidarity failures based on their lived experiences and as per their understandings of solidarity. These examples will ensure any eventual conceptual additions arising from research remain grounded in detailed context-specific examples.

### Analysis

Inductive and deductive thematic analysis will be conducted concurrently with interviews and completed in two stages.^53^ The first stage will involve analysis of interviews from the three participant categories. The last stage will involve identifying and discussing the significance of similarities, differences or any other important particularities emerging when responses from different participant categories are compared. Particular attention will be paid throughout the analytical stage to (1) imaginings of what more solidarity in/for GH should look like and require (2) potential points of convergence in statements about what and why solidarity is important or limited in the eyes of participants. In considering and comparing participant accounts, we will avoid using language or lenses of equivalence. Given our goal of thinking with and for a Pluriverse, it will be crucial to maintain recognition for differences surfaced.

For stage one focused on attending to experiences, meanings, perceived value (or limits) of current or otherwise imagined solidarity in and for GH in the eyes of specific categories of participants, three analysis teams will be struck. These will work independently on analysis of interviews from one participant group. Each of these analyst teams will be led by the PI (EN) with involvement of 2–4 experienced researchers on the team (co-PIs and senior researcher on staff), as well as 1–2 trainees. Inductive analysis will begin with PIs involved immersing themselves in the same 3–4 transcripts, to independently identify common language, themes, as well as particularities or differences that strike the analyst as significant in this set of transcripts. Through discussion, consensus will be reached on the structure of an initial codebook with key themes and sub-themes, reflecting these initial observations. Next, all transcripts from a participant category will be uploaded to Nvivo 15, and senior researcher(s) on the team, under the supervision of the study PI (EN) and with support from trainee(s) as appropriate, will iteratively code all transcripts. The PI and coding team will meet bi-weekly to discuss and reach agreement on additional emerging codes, or revisions to the codebook, consulting with other involved PIs as needed. The PI who will be familiar with all transcripts will regularly review the codebook for completeness and consistency in coding.

Upon completion of a participant category’s coding, a member of the study team external to those engaged in analysis for a participant category will review the definition and content of themes and sub-themes, to provide additional verification for clarity and consistency. The significance of emerging patterns and differences in participant responses, and points to emphasise in the write-up of findings, will then be completed in an analysis meeting with all members of a participant category analysis team. The above process will be guided by the Consolidated criteria for Reporting Qualitative research (COREQ) checklist for reporting qualitative research to ensure reliability, consistency and completeness.^54^

Once discrete analysis of the three participant groups is completed, all co-investigators, senior researchers and trainees will meet to discuss and reach consensus on how to best describe differences, similarities and particularities across participant categories.

Pragmatically, a challenge we anticipate in coding, analysis and refining of findings is that a pluriversal approach requires resisting a conventional analysis and representation of findings as indicative of ‘shared’ meaning. In our understanding of a pluriversal approach to qualitative research, space should not be left for participants’ accounts of the world and recommendations for action potentially not being amenable to consensus. Doing justice to the multiplicity of our participants’ worlds and perspectives while nevertheless pulling out implications for solidarity in and for GH will most likely require careful attention to how we represent our findings.

### Impacts of our positionality as researchers on and throughout the study

As researchers working within the GH ecosystem, we recognise that we are not neutral observers but active participants in the system we seek to interrogate. Our research team members self-identify as residents in both high-income countries and LMICs and include trainees (undergraduate, graduate, post-doctoral) as well as junior and senior academics. We are affiliated with universities and organisations that receive GH funding from major donors, and this project is funded and of interest to the top funder of GH research in the world: the Wellcome Trust. It is not lost on us that the decision of Wellcome to fund this project reinforces that organisation’s identity as committed to ‘Science to solve the urgent health challenges facing everyone’.^55^

Several team members have collaborated regularly with major funding agencies and implementing partners in the past, including WHO, United Kingdom Research and Innovation (UKRI), the European Commission and Wellcome. These positionalities offer both opportunities and risks. As a team, we do have first-hand experience with GH governance and funding dynamics. This proximity, coupled with the GH research programmes we lead, has alerted us to increasing mention of solidarity as a normative imperative in the sector in past years, alongside a lack of clear efforts to translate calls for solidarity into specific actions, different ways of operating and measurable impacts. We know and are ready to leverage the power we have, including our connections, understanding of what counts as compelling evidence and our expertise based on conventional, exclusionary and colonial norms for defining who gets trusted to conduct and report on research, but we also know this will normalise power/knowledge hierarchies within the GH ecosystem, even as we critique them. Some may argue there is a risk that, given our ongoing collaborations with GH funding and implementation organisations, we might temper our critiques, due to concerns about professional relationships or in anticipation critiques could limit our opportunities for future funding. We take such concerns and risks seriously and will make deliberate efforts to guard against these. First, we are approaching interviews with grant-makers and policy actors with clear protocols to separate data collection from advocacy or professional networking. Interviewers will disclose the academic purpose of the research, clarify that participation will not influence future funding decisions or collaborations and remind participants that they may decline to answer any questions. We will ensure that no team members interview participants with whom they have a direct funding or reporting relationship. We recognise that our interpretations will inevitably reflect our perspectives and locations within the GH ecosystem. Throughout the study, we will hold reflexivity sessions to critically examine how our social locations, disciplinary training and institutional affiliations may shape interview interactions, interpretation of findings and framing of recommendations. Moreover, we will ensure we include a reflexive commentary on our own locations within GH knowledge production in all publications or reports, in the interests of transparency. Our aim is not to erase this influence but to make it explicit and to intentionally foreground participants’ narratives and worldviews in analysis and dissemination.

### Patient and public involvement

This study is designed to foreground under-represented perspectives in GH, with most interviews (≅60) being conducted with community-level leaders in CSOs from all WHO regions working on distinct areas of focus in GH. All participants will be offered a copy of the study findings report in English, French or Spanish.

## Ethics and dissemination

### Dissemination and data sharing policy

This study will comply with the Wellcome Trust Data Sharing Policy. Findings from this study will be made available to the public at a minimum through webinars, peer-reviewed publications and through a brief trilingual report (French, Spanish, English). The research methodology will also be made available through Open Science Framework (OSF) and published in a peer-reviewed journal to provide other researchers access to the full methodological consideration and data collection and analysis steps. Following the Wellcome Trust Data Sharing Policy, there are no prohibitions on the reuse, sharing or distribution of the data from this study. As such, there are no copyright or licensing agreements associated with the data generated from this study. However, while publicly shared data in publications are available for public use, we will share de-identified data with researchers only on request submitted to the PI, which will be reviewed and approved by the entire team of investigators.

### Ethical consideration and discontinuation

Once participants confirm interest in participating in the study, letters of information and consent will be emailed to the participants. The letter of information and consent will detail the participant’s rights and what they should expect from the interview and will normally be signed and returned to the lead interviewer before the scheduled interview. In the case a participant is not able to return a signed copy of the consent form to us by email before their interview, consent will be sought verbally before an interview begins, with signed consent accepted after. During the study, participants may withdraw from the study before the study completion if they decide to do so at any time and for any reason. The research team also reserves the right to terminate a participant from the study if the team finds evidence of potential risk to the participant or that a participant is deliberately providing false information to influence the study result.

### Expected outcomes/discussion

This study aims to build understanding of how solidarity is understood and valued (or not) in and for GH. It does so with a particular interest in ensuring representation of actors who are not normally included at the tables of major GH funders and agenda-setting organisations, and with a particular interest in surfacing differences in perspectives from diversely positioned actors within the GH ecosystem. We are committed to engaging with and incorporating voices that might diverge or dissent from mainstream narratives. We also expect that consensus will likely remain elusive. By bringing diverse accounts of solidarity in and for GH into dialogue and debate, findings from this study can complement more abstract discussions of what solidarity does, can do, or should do for GH with examples of what it involves, what it produces, under what conditions, with what limitations, in the eyes of people who are engaged in GH programmes and projects on a day-to-day basis.

A primary outcome of this study will be a report, published on our project’s website in French, English and Spanish, that will detail how solidarity is conceptualised and attached to practices and conditions for realisation in similar or distinct ways by the three groups of actors we are interviewing: community-level leaders in CSOs working on GH issues; research institute directors in the Global South; and individuals with experience of funding decision-making with major GH funding and agenda setting organisations. This analysis may indicate significant variety, and also potentially some areas of alignment, in ways of identifying or thinking about what solidarity involves. Enriching understandings of solidarity in this way can facilitate more nuanced, more widely meaningful and more actionable commitments to solidarity by those who wish to align themselves with this term in GH. Our findings will also inform, alongside findings from reviews of the literature and regional workshops, the design of a tool that could be used by GH funders to demonstrate and translate into specific ways of funding and working, their commitments to solidarity.

### Study limitations and challenges

This study involves interviews with ≅ 100 GH actors. While we expect to be able to identify and explain points of resonance and difference in meanings and practices of solidarity across and within participant groups, the anticipated and intentionally sought variety in participant characteristics in terms of area of GH work, position in organisations, community-level engagement, gender and WHO region means findings will not be amenable to conclusions about what solidarity in a particular locality or for a particular population.

We are inviting potential participants to complete interviews in the language of their choice. Our interview team masters a fraction, eight, of the world’s languages. Our plan is to secure skilled interpreters and high-quality transcription and translation for interviews beyond our team competencies as needed. This approach, while removing barriers to participation, comes with the challenge of relying on translations for which no one on the team can confirm are capturing participants’ full intended meaning. It may also result in additional complexities for analysis if participants opt for languages where there is no translation for solidarity.

Data collection and analysis will involve a team dispersed across multiple countries and inclusive of several trainees. This approach will add complexity to interview and analysis processes. While the PI will be listening to every team member’s first interviews to confirm consistency and completeness in use of the interview guide and suggest areas for improvement as needed before a team member pursues subsequent interviews, it is anticipated there will be some variation across the interviews conducted. How much and what a participant shares will be influenced by participant-interviewer dynamics that will be even more variable across interviews with multiple interviewers involved. Different members of the interview team will bring different positionalities, disciplinary lenses and training experiences to the analysis. With multiple team members coding interviews, additional labour will be involved in holding meetings to collaboratively establish and advance codebooks, and to ensure consistency in the application of agreed-upon themes to the data.

## Supplementary material

10.1136/bmjopen-2024-095243 online supplemental file 1

10.1136/bmjopen-2024-095243 online supplemental file 2

10.1136/bmjopen-2024-095243 online supplemental file 3
