# Supplementary material for: Meanings and practices of solidarity in global health: a qualitative investigation - study protocol
Source: BMJ Open. 2026 Jan 7;16(1):e095243. doi: 10.1136/bmjopen-2024-095243 (PMC12781975; doi:10.1136/bmjopen-2024-095243)
Supplement: Supplementary file 4 [file bmjopen-2024-095243.reviewer_comments.pdf]

PEER REVIEW HISTORY

BMJ Open publishes all reviews undertaken for accepted manuscripts. Reviewers are asked to complete a checklist review form and are provided with free text boxes to elaborate on their assessment. These free text comments are reproduced below.

ARTICLE DETAILS

Title (Provisional)

Meanings and Practices of Solidarity in Global Health: A Qualitative Investigation - Study Protocol

Authors

Nouvet, Elysee; Ndu, Mary; Pratt, Bridget; Arguedas Ramirez, Gabriela; Prainsack, Barbara; Karunakara, Unni; DeVries, Jantina; Owusu, Samuel Asiedu; Atuire, Caesar

---

VERSION 1 - REVIEW

**Reviewer**	**1**
**Name**	**Leaune, Edouard**
**Affiliation**	**Université Claude Bernard Lyon 1**
**Date**	**21-Jan-2025**
**COI**	**None**

I would like to thank the authors for the opportunity to read their very interesting protocol paper. I think that this paper is of great interest and suitable for publication after minor revisions.

1. It would be interesting to add information on the background of the authors to address the potential biases in their future analysis of the data.

2. It would be interesting to clarify how some issues related to translation between three languages are addressed. Indeed, some terms may have different meanings in English, Spanish or French, and this will need to be addressed by the research team.

3. The limitations of the study should be more clearly described.

4. The strengths and limitations of working as an international team should be clearly addressed.

**Reviewer**	**2**
**Name**	**Gram, Lu**
**Affiliation**	**UCL Institute for Global Health**

**Date**	**04-Aug-2025**
**COI**	**None**

Overall comments

The study is a timely and long overdue study on conceptualisations of and enablers and barriers to solidarity in the Global Health ecosystem. It will draw on 100 interviews with 1. community-level leaders in civil society organizations working on GH issues; 2. research institute directors in the Global South; and 3. individuals with experience of funding decision-making with major GH funding and agenda setting organizations ("GH influencers"). The strengths of the study are that it is well-motivated with a strong rationale, a clear evidence gap, a novel theoretical framework (pluriversality), and clearly defined protocol for analysis. I have a few suggestions for areas of improvement that I think could strengthen it though:

• Clarity around the qualitative methodological approach

• Rooting the theoretical framework more solidly in theories of politics

• Providing more specificity around sampling

• Declaring and discussing the positionality of the authors themselves

Overall, it is a well-designed piece of research that definitely deserves to be carried out. Many have suffered from unequal and unjust power dynamics in Global Health and having a piece that examines how we can build solidarity-based practices to combat this is long overdue.

Major points

Greater clarity around the qualitative methodological approach is needed. Right now, the only overall description of study type is "qualitative study." But what kind of qualitative study is it? Is it a narrative study? A grounded theory study? A case comparison study? An ethnographic study? A phenomenological study? All qualitative are obviously not the same and the authors need to be explicitly about what kind of qualitative study it is, so that readers can understand what kinds of ontological and epistemological assumptions have been made and where the design choices for the study methods come from.

The theoretical framework theorises power, but does not confront it. At the level of individual communities, where members live in close proximity, e.g. a village or an urban neighbourhood, and have repeated interactions with one another based on trust and reciprocal exchange, collective action is much more frictionless than nation states competing for power, resources, and influence on a geopolitical stage. There is almost an underlying assumption that as long as the Global Health community realises how wrong we have been about our way of furthering health in the Global South, that will translate into more genuine solidarity globally. But the examples brought up by the authors themselves – COVID-19

vaccine nationalism, funding and prestige being attached to Global North institutions, climate change inaction – are not due to a lack of understanding of what solidarity means, but an active choice by people in power not to practice a solidarity that they understood extremely well. To give a clear example of this, that the Pentagon and the CIA decided to run an anti-vaccine misinformation campaign in the Philippines at the height of COVID-19 has nothing to do with misunderstanding solidarity:

https://academic.oup.com/jpubhealth/article/46/4/e685/7718885

We see the same happening with the rollback of USAID and global aid funding in Europe and so on. I think the authors need to expand their theoretical framework to engage properly with politics on the international arena and incorporate theories from international relations, committing to a particular framework whether realist, constructivist, liberal, or Marxist, etc. and thinking through how power intersects with solidarity. Otherwise, the authors risk simply reproducing known truisms like nations act in their own self-interest and undermine solidarity.

Sampling and recruitment methods need greater specificity. Although it is useful to keep a sampling frame relatively open-ended in qualitative research, a study on solidarity in Global Health would benefit from explicitly committing to a few details up front. In particular, which countries and/or world regions will be involved? Although the authors might not be able to list all countries involved, it would help if they could list a few example countries, so the reader has an idea of where in the world the study will be carried out. In particular, I am concerned about the fact that data collection will be entirely limited to interviews carried out in Spanish, French, and English – dominant European languages with colonial history – when the project itself was supposed to decolonise Global Health. The authors specify that they will represent all WHO regions, but WHO regions are enormous (Southeast Asia or Western Pacific) and lump wildly disparate countries (India and Thailand or China, Samoa, and Australia) together. For a study on solidarity in Global Health, it may be quite important to think carefully through how credible representation of the different regions of the world will be achieved.

Author positionality deserves reflection. As Global Health researchers, the authors are also part of the same Global Health ecosystem, they seek to critique, and directly or indirectly benefit from or suffer from the same system. As such, it is critical to take authorial positionality seriously – this goes beyond a biographical statement. The protocol should explicitly discuss how the research team's own relationships with funders, partners, and institutions under study will be managed. For example, how will they mitigate power asymmetries when interviewing grant-makers or policy actors with influence over their own careers or institutions? Being in the room with globally influential actors can carry an emotional charge — a mix of awe, admiration, or even the thrill of proximity to power. What strategies will ensure that the analysis does not become unduly awe-struck or deferential towards figures of power?

Minor points

Sampling and recruitment methods could also benefit from clarity on these questions:

o How will you access key stakeholders to be interviewed?

o Which types of non-community organisations will you approach?

VERSION 1 - AUTHOR RESPONSE

Reviewer: 1
Dr. Edouard Leaune, Université Claude Bernard Lyon 1
Comments to the Author:
I would like to thank the authors for the opportunity to read their very interesting protocol paper. I think that this paper is of great interest and suitable for publication after minor revisions.

1. It would be interesting to add information on the background of the authors to address the potential biases in their future analysis of the data.

*Excellent point. This has been added at the end of Methods section (pp. 12-13).

Impacts of our positionality as researchers on and throughout the study

As researchers working within the global health (GH) ecosystem, we recognize that we are not neutral observers but active participants in the system we seek to interrogate. Our research team members self-identify as residents in both high-income countries (HICs) and low- and middle-income countries (LMICs), and includes trainees (undergraduate, graduate, post-doctoral) as well as junior and senior academics. We are affiliated with universities and organizations that receive GH funding from major donors, and this project is funded and of interest to the top funder of GH research in the world: the Wellcome Trust. It is not lost on us that the decision of Wellcome to fund this project reinforces that organization's identity as committed to "Science to solve the urgent health challenges facing everyone" (https://wellcome.org/).

Several team members collaborate regularly with major funding agencies and implementing partners in the past, including the WHO, UKRI, the European Commission, and Wellcome. These positionalities offer both opportunities and risk. As a team, we do have first-hand experience with GH governance and funding dynamics. This proximity, coupled with the GH research programs we lead, has alerted us to increasing mention of solidarity as a normative imperative in the sector in past years, alongside a lack of clear efforts to translate calls for solidarity into specific actions, different ways of operating, and measurable impacts. We know and are ready to leverage the power we have, including our connections, understanding of what counts as compelling evidence, and our expertise based on conventional, exclusionary, and colonial norms for defining who gets trusted to conduct and report on research, but we also know this will normalize power/knowledge hierarchies within the GH ecosystem, even as we critique them. Some may argue there is a risk that, given our

ongoing collaborations with GH funding and implementation organizations, we might temper our critiques, due to concerns about professional relationships or in anticipation critiques could limit our opportunities for future funding. We take such concerns and risks seriously, and will make deliberate efforts to guard against these. First, we are approaching interviews with grant-makers and policy actors with clear protocols to separate data collection from advocacy or professional networking. Interviewers will disclose the academic purpose of the research, clarify that participation will not influence future funding decisions or collaborations, and remind participants that they may decline to answer any questions. We will ensure that no team members interview participants with whom they have a direct funding or reporting relationship. We recognize that our interpretations will inevitably reflect our perspectives and locations within the GH ecosystem. Throughout the study, we will hold reflexivity sessions to critically examine how our social locations, disciplinary training, and institutional affiliations may shape interview interactions, interpretation of findings, and framing of recommendations. Moreover, we will ensure we include a reflexive commentary on our own locations within GH knowledge production in all publications or reports, in the interests of transparency. Our aim is not to erase this influence but to make it explicit, and to intentionally foreground participants' narratives and worldviews in analysis and dissemination.

2. It would be interesting to clarify how some issues related to translation between three languages are addressed. Indeed, some terms may have different meanings in English, Spanish or French, and this will need to be addressed by the research team.

** We have expanded our inclusion criteria, so that potential participants may speak any language. We have included a new paragraph, under Data collection (pp. 8-9), explaining this decision. This paragraph draws attention to the team's fluency in multiple languages and the concerns we discussed as a team with respect to working in languages in which we do not have fluency (including interpretation/analysis challenges and the inability to verify the accuracy of translations). In this paragraph we provide our approach to these challenges, and our intention to acknowledge this limitation in eventual study outputs, should participants opt to use languages in which we are not fluent (see below). We have acknowledge language-related challenges in the new Study limitations and challenges section (p. 14)*

Another key sampling decision centered on participant inclusion/exclusion based on language fluency. Given our pluriversal framework, and desire to centre under-heard perspectives, we understood the importance of offering to participants the opportunity to participate in interviews in the language of their choice. At the same time, the PI (EN) was concerned about her ability to interpret participant meanings accurately for interviews conducted in languages in which she is not fluent (Spanish, English, French), as well as concerns about our team's capacity to verify the accuracy of translations beyond the 9 languages we master as a team. In the end, a decision was made that we

would offer to conduct interviews in the language of participants' choosing, with plans to appropriately resource high-quality professional interpretation and transcription/translation. We also will acknowledge in publications the limitation of our analysis where/if we are relying on translations of interviews from languages in which the team is not fluent. Sampling and recruitment strategies for each of our three participant categories are described below.

3. The limitations of the study should be more clearly described.
*We have added a section "Study limitations and challenges" (p. 14), with our addition reading as follows:

We are inviting potential participants to complete interviews in the language of their choice. Our interview team masters a fraction, eight, of the world's languages. Our plan is to secure skilled interpreters and high-quality transcription and translation for interviews beyond our team competencies as needed. This approach, while removing barriers to participation, comes with the challenge of relying on translations for which no one on the team can confirm are capturing participants' full intended meaning. It may also result in additional complexities for analysis if participants opt for languages where there is no translation for solidarity.

Data collection and analysis will involve a team dispersed across multiple countries and inclusive of several trainees. This approach will add complexity to interview and analysis processes. While the PI will be listening to every team member's first interviews to confirm consistency and completeness in use of the interview guide, and suggest areas for improvement as needed before a team member pursues subsequent interviews, it is anticipated there will be some variation across the interviews conducted. How much and what a participant shares will be influenced by participant-interviewer dynamics that will be even more variable across interviews with multiple interviewers involved. Different members of the interview team will bring different positionalities, disciplinary lenses, and training experiences to the analysis. With multiple team members coding interviews, additional labor will be involved in holding meetings to collaboratively establish and advance codebooks, and to ensure consistency in the application of agreed-upon themes to the data.

4. The strengths and limitations of working as an international team should be clearly addressed.
*See above

Reviewer: 2
Dr. Lu Gram, UCL Institute for Global Health
Comments to the Author:
Overall comments

The study is a timely and long overdue study on conceptualisations of and enablers and

barriers to solidarity in the Global Health ecosystem. It will draw on 100 interviews with 1. community-level leaders in civil society organizations working on GH issues; 2. research institute directors in the Global South; and 3. individuals with experience of funding decision-making with major GH funding and agenda setting organizations ("GH influencers"). The strengths of the study are that it is well-motivated with a strong rationale, a clear evidence gap, a novel theoretical framework (pluriversality), and clearly defined protocol for analysis. I have a few suggestions for areas of improvement that I think could strengthen it though:

- Clarity around the qualitative methodological approach
- Rooting the theoretical framework more solidly in theories of politics
- Providing more specificity around sampling
- Declaring and discussing the positionality of the authors themselves

Overall, it is a well-designed piece of research that definitely deserves to be carried out. Many have suffered from unequal and unjust power dynamics in Global Health and having a piece that examines how we can build solidarity-based practices to combat this is long overdue.

Major points

Greater clarity around the qualitative methodological approach is needed. Right now, the only overall description of study type is "qualitative study." But what kind of qualitative study is it? Is it a narrative study? A grounded theory study? A case comparison study? An ethnographic study? A phenomenological study? All qualitative are obviously not the same and the authors need to be explicitly about what kind of qualitative study it is, so that readers can understand what kinds of ontological and epistemological assumptions have been made and where the design choices for the study methods come from.

**We thank the reviewer for this request for expanded explanation of our qualitative study design. We have added details of our commitments to an interpretive study design (also noted in abstract now), informed by an interpretivist paradigm. Here is the exact addition (p. 6):*

We are adopting an interpretive research design (Schwartz-Shea & Yanow, 2012), suitable to our intentions of exploring diverse participants' accounts of what solidarity means and involves in their view, with attention to how these accounts may differ based on participants' positions and experiences within a vast GH ecosystem. In accordance with the interpretivist paradigm at the core of interpretive research design (Guba & Lincoln, 1994), we are not approaching the main object of investigation in this study, solidarity, as something that can yield to fixed or universal representation. Instead, solidarity is regarded as a term whose meaning emerges in dynamic social processes within particular contexts, and depends on interpretation by particular actors whose social identities, relationships to language, structures of power, and biographically unique experience shape their understanding and application of this term (Guba & Lincoln, 1994; Braun & Clarke, 2021; Schwartz-Shea & Yanow, 2012).

Flexibility sits at the heart of interpretive research design. Interpretive research design invites attention to emerging themes, unexpected interpretations, and strives to expand understanding of social phenomena through a shuttling between existing theory and data (Braun & Clarke, 2019; Guba & Lincoln, 2004). Reflexivity is also central to this design. Recognizing that knowledge emerges within social processes and between distinctly situated human actors, this approach requires researchers to remain attentive to their

role as co-producers of knowledge with study participants, and critically consider the ways in which their own positionality shape their interpretation and representation of participants' narratives (Braun & Clarke, 2021; Yanow & Schwartz-Shea, 2006). More details on the research team's positionality and implications for our study procedures are provided at the end of the methods section.

The theoretical framework theorises power, but does not confront it. At the level of individual communities, where members live in close proximity, e.g. a village or an urban neighbourhood, and have repeated interactions with one another based on trust and reciprocal exchange, collective action is much more frictionless than nation states competing for power, resources, and influence on a geopolitical stage. There is almost an underlying assumption that as long as the Global Health community realises how wrong we have been about our way of furthering health in the Global South, that will translate into more genuine solidarity globally. But the examples brought up by the authors themselves – COVID-19 vaccine nationalism, funding and prestige being attached to Global North institutions, climate change inaction – are not due to a lack of understanding of what solidarity means, but an active choice by people in power not to practice a solidarity that they understood extremely well. To give a clear example of this, that the Pentagon and the CIA decided to run an anti-vaccine misinformation campaign in the Philippines at the height of COVID-19 has nothing to do with misunderstanding solidarity:
https://academic.oup.com/jpubhealth/article/46/4/e685/7718885
We see the same happening with the rollback of USAID and global aid funding in Europe and so on. I think the authors need to expand their theoretical framework to engage properly with politics on the international arena and incorporate theories from international relations, committing to a particular framework whether realist, constructivist, liberal, or Marxist, etc. and thinking through how power intersects with solidarity. Otherwise, the authors risk simply reproducing known truisms like nations act in their own self-interest and undermine solidarity.

**We are grateful for this observation, as we can see how the previous version did seem to say we assumed more understanding would/could lead to solidarity being enacted more effectively in and for GH. We have made additions in the introduction, citing the work of Arguedas (2025) and Klein (2008), to explicitly acknowledge that current failures of solidarity are not accidental, but political and baked into the current system:*

Failures to enact solidarity globally are not accidental. Ours is an era of normalized tolerance towards structural injustices, linked to colonial and imperial legacies, and a dominant neoliberal capitalist world order (Arguedas, 2025). The dominant ethos for political and economic engagements in this order is competition, not collaboration. Countries in the Global North and big corporations that profit from their dominance in this order do gain more, profit- and power-wise, by maintaining structural injustices and leveraging the business opportunities these afford, than working to transform them (Arguedas, 2025; Klein 2008). It is not surprising against this backdrop, that the rhetoric of GH solidarity often does not match the reality of GH governance and action.

**Pluriversality and the critical interrogation of solidarity in GH it affords proposes methods for social (re)design. In particular, it calls for attention in research to what participants say they desire and what may be possible beyond the current system (Leitão, 2022) . We have revised the pluriversality section under study design, to make this more apparent. We also close that section specifying that we regard buy-in from those with more power in the GH*

ecosystem as crucial to insights from underheard and community-level actors having any influence on dominant norms of practice, a point also re-iterated in our details on data collection with "Influencers".

New paragraph in pluriversality (study design) section:

We are aware that centering under-recognized and in particular community-level experiences, meanings, and ways of working, is insufficient in and of itself to render solidarity more actionable at a global level. As noted in the introduction, GH work exists within a dominant world order premised on competition and hierarchies of power, entrenched through colonialist and imperialist legacies, and organized to reproduce itself, inequities, and its ways of working. If insights from this study are to impact on practice at the global level, our team's view is this requires buy-in from big players in the ecosystem. This underlies our decision as a team to make a key output of the broader research program within this study a tool to guide GH funders aiming to translate their commitments to solidarity into funding policies and practices.

Sampling and recruitment methods need greater specificity. Although it is useful to keep a sampling frame relatively open-ended in qualitative research, a study on solidarity in Global Health would benefit from explicitly committing to a few details up front. In particular, which countries and/or world regions will be involved? Although the authors might not be able to list all countries involved, it would help if they could list a few example countries, so the reader has an idea of where in the world the study will be carried out. In particular, I am concerned about the fact that data collection will be entirely limited to interviews carried out in Spanish, French, and English – dominant European languages with colonial history – when the project itself was supposed to decolonise Global Health. The authors specify that they will represent all WHO regions, but WHO regions are enormous (Southeast Asia or Western Pacific) and lump wildly disparate countries (India and Thailand or China, Samoa, and Australia) together. For a study on solidarity in Global Health, it may be quite important to think carefully through how credible representation of the different regions of the world will be achieved.

**Thank you for this comment, which we have used to strengthen our methods.*

Three key suggestions/concerns are contained in the above comment: a suggestion for more detail on recruitment and sampling; concern that our study will not achieve credible representation of different regions of the world; and, concern with the conduct of interviews in three languages only, all of which are "dominant European languages with colonial history". Below, we outline how the revised manuscript addresses each of these concerns.

Suggestion 1: more detail on recruitment

**We have revised the Data collection section to add detail on identification and recruitment for each participant category (pp. 7-10)*

Concern 2: credible representation and details on countries from which participants recruited.

**Our sampling is designed for optimal diversity and our recruitment across WHO regions is tied to this objective only. We have now included a statement to render more explicit what sampling across WHO regions will involve in practice and aim to achieve (p. 9).*

We will aim to recruit participants across all WHO regions, and from diverse countries within those regions, but we will not be interpreting or comparing participant responses based on their location at birth or at time of interview. Indeed, such analysis would almost certainly involve making very problematic assumptions, given our sample size, about how participant's perspectives on solidarity connect to regional or national influences. We are recruiting across WHO regions and diverse countries towards maximizing potential for variation in participant perspectives.

Concern 3: concern with the conduct of interviews in three languages only, all of which are "dominant European languages with colonial history".

**We have adapted our interview plan to include any language participants may choose, and added on page 8 details on what was considered in this decision. The Limitations and challenges section (new, pp. 14-15) also acknowledges potential challenges/limitations associated with reliance on interpretation/translations for interviews conducted in languages in which our team is not fluent.*

Another key sampling decision centered on participant inclusion/exclusion based on language fluency. Given our pluriversal framework, and desire to centre under-heard perspectives, we understood the importance of offering to participants the opportunity to participate in interviews in the language of their choice. At the same time, the PI (EN) was concerned about her ability to interpret participant meanings accurately for interviews conducted in languages in which she is not fluent (Spanish, English, French), as well as concerns about our team's capacity to verify the accuracy of translations beyond the eight languages we master as a team. In the end, a decision was made that we would offer to conduct interviews in the language of participants' choosing, with plans to appropriately resource high-quality professional translation. We also will acknowledge in publications the limitation of our analysis where/if we are relying on translations of interviews from languages in which the team is not fluent. Sampling and recruitment strategies for each of our three participant categories are described below.

Author positionality deserves reflection. As Global Health researchers, the authors are also part of the same Global Health ecosystem, they seek to critique, and directly or indirectly benefit from or suffer from the same system. As such, it is critical to take authorial positionality seriously – this goes beyond a biographical statement. The protocol should explicitly discuss how the research team's own relationships with funders, partners, and institutions under study will be managed. For example, how will they mitigate power asymmetries when interviewing grant-makers or policy actors with influence over their own careers or institutions? Being in the room with globally influential actors can carry an emotional charge — a mix of awe, admiration, or even the thrill of proximity to power. What strategies will ensure that the analysis does not become unduly awe-struck or deferential

towards figures of power?

**Thank you for this observation and suggestion, and we completely agree this is worth including. Our research team actually discusses this at every meeting, are planning an end-of-study piece reflecting on these power dynamics and our positionality, and are very happy to include this in the protocol. We have added the following to the end of our methods section (pp. 12-13)*

Impacts of our positionality on and throughout the study

As researchers working within the global health (GH) ecosystem, we recognize that we are not neutral observers but active participants in the system we seek to interrogate. Our research team members self-identify as residents in both high-income countries (HICs) and low- and middle-income countries (LMICs), and includes trainees (undergraduate, graduate, post-doctoral) as well as junior and senior academics. We are affiliated with universities and organizations that receive GH funding from major donors, and this project is funded and of interest to the top funder of GH research in the world: the Wellcome Trust. It is not lost on us that the decision of Wellcome to fund this project reinforces that organization's identity as committed to "Science to solve the urgent health challenges facing everyone" (https://wellcome.org/).

Several team members collaborate regularly with major funding agencies and implementing partners in the past, including the WHO, UKRI, the European Commission, and Wellcome. These positionalities offer both opportunities and risk. As a team, we do have first-hand experience with GH governance and funding dynamics. This proximity, coupled with the GH research programs we lead, has alerted us to increasing mention of solidarity as a normative imperative in the sector in past years, alongside a lack of clear efforts to translate calls for solidarity into specific actions, different ways of operating, and measurable impacts. We know and are ready to leverage the power we have, including our connections, understanding of what counts as compelling evidence, and our expertise based on conventional, exclusionary, and colonial norms for defining who gets trusted to conduct and report on research, but we also know this will normalize power/knowledge hierarchies within the GH ecosystem, even as we critique them. Some may argue there is a risk that, given our ongoing collaborations with GH funding and implementation organizations, we might temper our critiques, due to concerns about professional relationships or in anticipation critiques could limit our opportunities for future funding. We take such concerns and risks seriously, and will make deliberate efforts to guard against these. First, we are approaching interviews with grant-makers and policy actors with clear protocols to separate data collection from advocacy or professional networking. Interviewers will disclose the academic purpose of the research, clarify that participation will not influence future funding decisions or collaborations, and remind participants that they may decline to answer any questions. We will ensure that no team members interview

participants with whom they have a direct funding or reporting relationship. We recognize that our interpretations will inevitably reflect our perspectives and locations within the GH ecosystem. Throughout the study, we will hold reflexivity sessions to critically examine how our social locations, disciplinary training, and institutional affiliations may shape interview interactions, interpretation of findings, and framing of recommendations. Moreover, we will ensure we include a reflexive commentary on our own locations within GH knowledge production in all publications or reports, in the interests of transparency. Our aim is not to erase this influence but to make it explicit, and to intentionally foreground participants' narratives and worldviews in analysis and dissemination.

Minor points

Sampling and recruitment methods could also benefit from clarity on these questions:
o How will you access key stakeholders to be interviewed?

o Which types of non-community organisations will you approach?

We have slightly re-organized and made minor edits in the Data collection section to render more explicit our strategy vis a vis non-community organizations. We have added headers to draw attention to our description of sampling and recruitment for different participant categories, and this now identifies one group as "community-level participants" (and thus the others as not, without saying they are not).

We have also added at the outset of Data collection section the following explanation of our rationale and insight on our team's decision-making processes related to including community-level and non-community level actors (p. 8):

Deciding on who to seek out for interviews, and whether or not to actively recruit different types of actors in the GH ecosystem, was not without debate. Some on our team felt strongly that our pluriversal framework should translate into centering insights and experiences of historically and conventionally excluded or lesser heard GH actors. Disrupting norms of GH discourse that reproduce the authority of certain worlds and knowledges while excluding others, requires intentionally seeking out and asserting the validity of alternative perspectives. Based on this rationale, all participants might be recruited from amongst a category of actors that conventionally holds limited power over global health fundings and other discourses: actors working on GH issues at the community level. Ultimately a decision was made to expand recruitment beyond this basis for inclusion, based on two logics. First, hearing from both community-level actors and actors who do not work primarily at the community level, might render more apparent important particularities to ways of thinking about and enacting solidarity, depending on whether participants worked primarily at the community or non-community level. The insights from community-level participants might thus become more apparent to us as a research team, and easier to describe in our analysis, through comparison to empirical data from interviews with otherwise positioned global health

actors. Secondly, it was agreed that our team could not and should not ignore that GH research institute directors and major funding agencies hold central (though certainly distinct) roles in enacting or interrupting norms of practice in the sector. Gathering, analyzing, and rendering available for broader consideration reflections on solidarity from these actors, might illuminate important considerations for rendering community-level actors' insights on solidarity more actionable at non-community levels.
*****

Reviewer: 1
If you have selected 'Yes' above, please provide details of any competing interests.: Not applicable

Reviewer: 2
If you have selected 'Yes' above, please provide details of any competing interests.: Not applicable

---

VERSION 2 - REVIEW

**Reviewer**	**1**
**Name**	**Leaune, Edouard**
**Affiliation**	**Université Claude Bernard Lyon 1**
**Date**	**22-Oct-2025**
**COI**	

The authors addressed all of my comments adequately.

**Reviewer**	**2**
**Name**	**Gram, Lu**
**Affiliation**	**UCL Institute for Global Health**
**Date**	**08-Nov-2025**
**COI**	

The authors have addressed most of my concerns. My one remaining concern is with the discussion of power in their protocol - they focus on coloniality, globalization, and multinational capital, but I think they fail to respond to the present moment, if they leave out the impacts of rising ethno-nationalism and the retreat of multilateralism that is key ton our present moment. For example, the current rollback of USAID is not premised on a capitalist logic, but rather than ethno-nationalist logic. Failure in their analysis to account for

the fragmentation of global multilateralism - an institutional infrastructure for global solidarity in the first place - would again risk making it outdated for current times, so I would strongly encourage the authors to include this.

VERSION 2 - AUTHOR RESPONSE

Reviewer: 2
Dr. Lu Gram, UCL Institute for Global Health
Comments to the Author:
The authors have addressed most of my concerns. My one remaining concern is with the discussion of power in their protocol - they focus on coloniality, globalization, and multinational capital, but I think they fail to respond to the present moment, if they leave out the impacts of rising ethno-nationalism and the retreat of multilateralism that is key ton our present moment. For example, the current rollback of USAID is not premised on a capitalist logic, but rather than ethno-nationalist logic. Failure in their analysis to account for the fragmentation of global multilateralism - an institutional infrastructure for global solidarity in the first place - would again risk making it outdated for current times, so I would strongly encourage the authors to include this.

Please find below our response to the 1 comment / suggestion for (minor) revision by Dr. Lu Gram:
We have added acknoweldgement of ethno-nationalism and the retreat of multilateralism to our framing of the current world order in the introduction. We disagree with Dr. Gram in their opinion that the crisis of multilateralism is something different or unrelated to the function of neoliberal capitalism, so we have not presented multilateralism as separate from neoliberal capitalist logic, and instead rendered explicit that we see these things as connected. The logic of neoliberal capitalism, in its most extreme form (what philosopher, theologian and economist Franz Hinkelammert called modernindad in extremis), implies the elimination of any form of solidaristic governmental policy. Multilateralism can only survive if there are political convictions rooted in a shared sense of humanity and common good. Neoliberal capitalism in extremis rejects that because that approach (multilateralism) imposes limits and regulations on the functioning of markets and businesses. disagree with him in his opinion that the crisis of multilateralism is something different or unrelated to the function of neoliberal capitaism. The only multilateral institutions and systems that are survivigin right now are those deidcated to protect the interestes and necesities of big businesses, such as intellectual property rigths, trade agreements, etc. While we acknowledge now in the protocol the fragmentation of multi-lateralism and rise of ethno-nationalism, we frame these as connected to current dominant capitalist logic.
